# The Impact of Total Intravenous Anesthesia and Volatile Anesthetics on Minimizing Cancer Recurrence and Postoperative Cognition

**DOI:** 10.7759/cureus.87379

**Published:** 2025-07-06

**Authors:** Issac H Jin, Michael W Lew

**Affiliations:** 1 Anesthesiology, Drexel University College of Medicine, Philadelphia, USA; 2 Anesthesiology and Perioperative Medicine, City of Hope National Medical Center, Duarte, USA

**Keywords:** cancer recurrence, onco anesthesia, postoperative cognitive dysfunction, postoperative delirium, sevoflurane vs propofol

## Abstract

As the incidence of cancer and the number of annual oncologic surgeries continue to rise, especially among younger populations, there is increasing interest in how perioperative factors, including the choice of anesthetic agent, may influence long-term oncologic survival outcomes and postoperative cognitive recovery. While surgery remains a mainstay component of treatment for solid organ malignancies, the physiological stress and inflammatory response triggered during the perioperative period may play a meaningful role in tumor progression, recurrence, and metastasis. Given these concerns and the potential benefit of selective anesthesia, the choice of anesthetic agent has gained increased attention for its potential to impact patient care, recovery, and outcomes beyond perioperative management, including postoperative delirium (POD) and postoperative cognitive dysfunction (POCD). In addition to providing adequate intraoperative sedation, propofol-based total intravenous anesthesia (TIVA) and inhaled volatile agents have been studied with the intent of characterizing their oncologic impact, which has yielded mixed results, making it difficult to conclude that one agent is superior to the other. Limitations in trial design, cancer heterogeneity, and confounding perioperative factors, such as agent administration timing and adjunctive sedation, underscore the need for larger-scale, multicenter randomized trials with long-term follow-up periods to better characterize the impact of anesthetic agents. Until more definitive evidence emerges, anesthetic choice should be individualized based on individual comorbidities, cognitive risk factors, surgical context, and anesthesiologist familiarity.

## Introduction and background

Cancer continues to reign as one of the leading causes of premature death around the world [1]. By 2050, 35.3 million cancer cases are expected worldwide, a 76.6% increase from the 2022 estimate, and 18.5 million cancer deaths are estimated by 2050, an 89.7% increase from the 2022 estimate [2]. The number of cancer cases with an indication for surgery is expected to increase by five million procedures globally between 2018 (9,065,000) and 2040 (13,821,000) [3]. Cancer patient demographics are also increasingly shifting toward middle-aged populations [4], indicating an earlier average age of cancer diagnosis and potentially having a substantial impact on employment, productivity, and expected healthcare costs among a younger patient population. Given the growing number of cancer cases among a younger generation, it is imperative to scrutinize the long-term effects of surgical trauma, physiologic stress, and immune suppression that may come with any major oncological surgical procedure [5] and the potential perioperative impact of anesthetic agents in reducing inflammation, enhancing immune function, and reducing postoperative complications during this particularly vulnerable period.

As surgery remains a central treatment modality for solid organ cancers, perioperative interventions, including anesthesia, have been postulated to influence the surgical inflammatory response [6]. Thus, the choice of anesthesia in cancer surgeries has increasingly gained attention for its potential to influence both immediate surgical outcomes and long-term cancer prognosis. Anesthetic agents have been associated with various immunomodulatory properties, including decreased natural killer cell activity, immunosuppression, angiogenesis, and pro-inflammatory responses [7]. Specific anesthetic induction agents, such as propofol, have been associated with enhanced immune function by demonstrating tumor-suppressing activity, while others have demonstrated potential tumor-promoting behavior. Beyond providing comfort and safety during invasive surgical procedures, anesthetic agents combined with procedural techniques may influence the microbiological landscape in ways that affect tumor recurrence, postoperative cognitive dysfunction (POCD), and ultimately patient survival. Although, in practice, the choice of induction agent should be individualized, this review aims to add to the evolving discussion of how selection of anesthetic induction agent, including total intravenous anesthesia (TIVA) with propofol and volatile anesthetic agents, may both effect cancer recurrence and minimize major POCD, especially in an aging patient population one would expect of typical cancer populations.

## Review

TIVA with propofol

Propofol has become one of the most widely utilized anesthetic induction agents in modern anesthesia practice due to its rapid onset, short duration of action, and favorable emergence profile [8]. Propofol induces its effects primarily through modulation of gamma-aminobutyric acid (GABA-A) receptors but also inhibits N-methyl-D-aspartate (NMDA) receptors and voltage-gated sodium and calcium channels. Its use in TIVA is especially valued due to its potential systemic benefits in reducing postoperative nausea and vomiting, free radical scavenging, cardiac and neuroprotective effects, and immunoprotection following surgery [9]. Recently, increasing evidence has emerged suggesting propofol's extensive non-anesthetic properties as an immunomodulator in addition to its inhibition of tumor angiogenesis, invasion, and metastasis that may play an important role in improving oncologic outcomes by reducing postoperative tumor recurrence as an ancillary anti-metastatic agent in cancer patients undergoing surgery [10].

Propofol’s anti-metastatic and anti-tumorigenic biochemical properties have been repeatedly demonstrated in several preclinical studies in various cancer cell lines and pathways in vitro. In lung cancer cells, propofol inhibited malignant cell proliferation, migration, and invasion by modulating metabolic pathways and key proteins involved in the signaling pathways associated with tumor progression [11]. Specifically, propofol was found to downregulate glucose transporter 1 (GLUT1) and mitochondrial pyruvate carrier 1 (MPC1), two key regulators of cancer cell metabolism and energy production. By disrupting glucose uptake and impairing mitochondrial integrity, these transporters may be crucial in starving cancer cells by impairing energy production. Propofol was also shown to upregulate pigment epithelium-derived factor (PEDF) expression, further augmenting its tumor suppressive effect by decreasing the malignancy of lung cancer cells. Propofol also suppressed small cell lung cancer growth and promoted apoptosis through the microRNA-21/phosphatase and tensin homolog/protein kinase B (miR-21/PTEN/AKT) signaling pathway in A549 non-small cell lung cancer (NSCLC) cells, both in vitro and in vivo in nude mice [12]. Propofol’s anti-tumor effect has also been demonstrated in colorectal cancer cell lines through its effect on the proliferation, migration, and invasion of human colorectal cancer cells in a dose-dependent manner [13]. In this cell line, propofol was found to exert an inhibitory effect by enhancing miR-124-3p.1 expression while reducing the expression of the pro-proliferative proteins cyclin D1 and metastasis-related proteins matrix metalloproteinase-9 (MMP-9) and vimentin. Propofol also appeared to have a similar effect on pancreatic cancer cell migration and invasion, with an enhancing effect on miRNAs involved in modulating cancer growth and proliferation, specifically miR328, to inhibit disintegrin and metalloproteinase 8 (ADAM8) [14].

Inhaled/volatile anesthetics

Inhaled anesthetics remain a foundational component in both induction and maintenance anesthesia. Volatile agents, such as sevoflurane, isoflurane, desflurane, and halothane, are extensively used in induction and maintenance in various surgical procedures [15]. Although their exact mechanism of action has not been fully elucidated, volatile agents are known to inhibit excitatory neurotransmission pathways through interactions with GABA-A, glycine, NMDA receptors, and voltage-gated sodium and calcium channels. Although used extensively in modern anesthesia, these agents' postoperative and oncologic implications remain unclear, and recent studies have produced mixed findings on their potential immunomodulatory effects.

Several in vitro studies have demonstrated the immunomodulatory effects of volatile anesthetics, particularly focusing on the effects of sevoflurane [15]. Sevoflurane has widespread familiarity and is used extensively in the clinical setting as maintenance anesthesia, given its ease of titration, rapid recovery, minimal airway irritation, and hemodynamic stability. In colon cancer cells, sevoflurane has been demonstrated to inhibit cell proliferation, induce apoptosis, and regulate epithelial-mesenchymal transition, potentially exerting an anti-tumor effect by regulating the extracellular signal-regulated kinase (ERK) signaling pathway [16]. Similarly, in hepatocellular carcinoma cell lines, sevoflurane exhibited anti-proliferative effects by downregulating the levels of microRNA-25-3p, demonstrating another potential pathway in anti-tumor activity [17]. At clinically relevant doses, sevoflurane was also shown to suppress the proliferation and migration of cervical cancer cells, although it did not significantly affect apoptosis or overall cell survival, and increased chemosensitivity, suggesting a potential role in augmenting cancer treatment efficacy in human cancer cell lines [18]. However, multiple studies have also suggested a potential tumorigenic effect of sevoflurane, indicating worse clinical outcomes in cancer patients. In cervical cancer cells, sevoflurane was found to upregulate histone deacetylase 6 (HDAC6) expression and to promote cell proliferation, migration, and invasion through PI3K/AKT and ERK1/2 signaling pathways [19]. In prostate cancer models, sevoflurane increased cancer cell invasion and lung metastasis by increasing macrophage M2 polarization through the Il-6/HO-1 pathway, again demonstrating its potential to facilitate cancer metastasis and worsen clinical outcomes [20]. In ovarian cancer cells, sevoflurane and desflurane were found to promote cell proliferation and migration through the miRNA-HIF-1α pathway, a known cancer-inducing factor [21]. The cancer-promoting effects were reversed upon introducing miR-138 and miR-210, suggesting the anesthetic's role in modulating key regulators in oncogenic processes.

Comparison of propofol and inhaled anesthetics

Several studies have compared the effects of propofol and inhaled volatile anesthetics on cancer recurrence and outcome. A meta-analysis of 20 studies, including three randomized controlled trials (RCTs) and 17 observational longitudinal studies, found that propofol-based TIVA was associated with a significantly lower risk of cancer metastasis and recurrence than inhalation anesthesia (INHA) [22]. Subgroup analysis demonstrated that propofol-based TIVA was associated with reduced metastasis in breast cancers, but not in other types of cancer. None of the three RCTs included in this meta-analysis showed a statistically significant difference in cancer metastasis or recurrence. However, the authors report limitations, as the study design of the Sessler 2019 study, the major RCT contributor to this meta-analysis, may have masked a potential effect by including patients with estrogen-positive breast cancer, which already has a favorable prognosis overall and was reflected in low recurrence rates in both groups [22]. Additionally, the two other RCTs included in this meta-analysis demonstrated a significant benefit of propofol-based TIVA over IHNA on patient outcomes, although the sample size was limited [22].

The impact of induction agent on postoperative inflammatory cytokine levels was also investigated postoperatively, building on the principle that major surgical trauma induces a systemic inflammatory response that includes increased IL-6 secretion and downstream pro-tumorigenic signaling pathways that are associated with increased invasiveness of metastasis [23]. While IL-6 is known to promote wound healing, it has also been shown to have pro-tumorigenic effects by stimulating cancer cell proliferation, invasiveness, and metastasis. Meta-analysis revealed that postoperative levels of IL-6 were significantly lower in patients receiving propofol-based TIVA compared to INHA, reflecting the efficacy of propofol's anti-inflammatory properties as previously discussed. Other pleiotropic cytokines, including TNF-α, also demonstrated a high predictive value for developing postoperative complications, including distant tumor metastasis, specifically in patients with primary, colorectal, and lung cancer surgeries [22].

A separate meta-analysis evaluated the survival benefit comparing propofol-based anesthesia and INHA in breast cancer patients [24] and found that patients who received propofol-based anesthesia demonstrated improved overall and recurrence-free survival. However, the authors acknowledged that limitations, such as short anesthesia duration during a brief perioperative period, mixed anesthesia, and neoadjuvant therapy, may have impacted these results. A retrospective study comparing propofol and sevoflurane in 2,838 patients undergoing radical breast and colorectal cancer surgeries with either propofol-based TIVA and sevoflurane-based anesthesia demonstrated a survival benefit for patients who received propofol at both one-year (4.7%) and five-year (5.6%) postoperative follow-ups [25]. However, this statistically significant survival benefit was lost when adjusted for confounders.

However, other studies have shown different outcomes. In an RCT that included 1,228 patients aged 65-90 years old undergoing major cancer surgery across 14 tertiary hospitals in China, patients were randomized to receive either propofol-based or sevoflurane-based INHA and had a median follow-up duration of 43 months [26]. The study found no statistically significant differences in long-term or recurrence-free survival in patients between propofol and sevoflurane-based groups (31% mortality and 29% mortality, respectively). Propofol did not improve cancer-related outcomes and even had slightly decreased scores for physical function on long-term follow-up. Additional subgroup analysis found a significant interaction between age and propofol, suggesting that patients older than 75 had worse overall survival with propofol than with sevoflurane. Additionally, a retrospective study involving patients who underwent esophageal cancer resection found no significant differences between propofol-based TIVA and INHA in perioperative inflammatory markers (neutrophil and platelet-to-lymphocyte ratios) and systemic immune-inflammatory index [27]. Altogether, these findings challenge the idea of a consistent inflammatory advantage associated with propofol and highlight the heterogeneity of outcomes across cancers, patient age, and surgical types.

Postoperative delirium (POD) and postoperative cognitive dysfunction (POCD)

POD continues to be one of the most common complications facing surgical recovery, particularly affecting older populations. As its prevalence increases with the severity of surgery, POD is expected to disproportionately affect patients undergoing major oncologic surgeries [28]. According to the Diagnostic and Statistical Manual of Mental Disorders, Fifth Edition (DSM-5), POD is defined as a disturbance in attention, cognition, or awareness that develops over a short period. It is characterized by a fluctuating course and can be associated with sedation and general anesthesia. The incidence of POD affects around one in five following elective surgery and approximately one in three following emergency surgery. It has also been associated with poorer surgical outcomes, including an increased risk of mortality, five times more likely to be discharged to a nursing facility, and 1.8 days longer hospital stay in non-cardiac, non-neurological surgical patients aged 60 and above [29]. This has primarily been considered a preventable complication that can be reduced through patient safety strategies, preoperative risk screening, medication review, frequent orientation, mobility, and enhanced sleep hygiene [30].

In contrast, POCD entails a subtle, long-term decline in specific cognitive domains following surgery, with onset of days to weeks postoperatively, and is characterized by impaired executive function, memory issues, decreased attention, and overall slower processing [31]. POCD requires pre- and postoperative neuropsychiatric testing for a precise diagnosis. Risk factors for POCD include advanced age, baseline cognitive or physical impairment, and longer, more complex surgeries. Like POD, POCD may resolve; however, a key distinction is that it may persist and lead to long-term dysfunction and negatively impact surgical outcomes and quality of life.

In a meta-analysis of 19 RCTs, different anesthesia methods (including general anesthesia, regional, and combination) were compared to determine the risk of POD in elderly patients. They found a significant reduction in POD risk between the combination of general and regional anesthesia groups compared to the general anesthesia group alone [32]. The study also concluded that peripheral nerve block with bupivacaine may help reduce rates, although the data are limited. Notably, no apparent risk reduction was found between anesthetic agents, including propofol and sevoflurane.

Previous studies have shown that propofol was associated with decreased POD and POCD, with the proposed mechanism being that it attenuates neuroinflammation and amyloid-B toxicity, leading to reduced cognitive dysfunction compared to volatile agents. Propofol-based TIVA and sevoflurane were directly compared in an RCT involving older patients undergoing major cancer surgery, and propofol-based TIVA was found to significantly reduce early POCD, particularly on postoperative day one, in this patient population [33]. These findings were also seen in adults undergoing coronary artery bypass surgery in a prospective RCT that compared propofol-based TIVA and sevoflurane anesthesia, which found that propofol-based TIVA was associated with a lower incidence of both POD and early POCD when compared to sevoflurane [34]. This difference was especially pronounced in patients older than 65 years old. In a separate study involving patients undergoing cardiopulmonary bypass, propofol was found to have better early cognitive function, faster anesthesia recovery, and less neuroinflammation, although lacking long-term POCD follow-up data [35].

Other studies have reported different results. In a single-center, double-blind RCT in 234 patients ages 65-86 undergoing elective tumor resection, there were no significant differences in POCD rates between propofol and sevoflurane at postoperative days seven and 30 [36]. Sevoflurane appeared to have a mild, transient adverse effect on early postoperative recovery quality, but did not affect long-term prognosis, as both groups performed similarly by three months postoperatively. A retrospective cohort study comparing various post-operative effects, including cognitive function, of propofol and sevoflurane as maintenance anesthesia in gastric cancer patients undergoing resection. Both cohorts, consisting of 40 patients, were induced with midazolam, sufentanil, and etomidate and were either maintained with TIVA with propofol or sevoflurane, depending on their assigned group. Compared to propofol, individuals who received sevoflurane had significantly elevated Loewenstein Occupational Therapy Cognitive Assessment (LOTCA) and the Mini-Mental State Examination (MMSE) scores, indicating less cognitive impairment [37].

## Conclusions

Both propofol-based TIVA and sevoflurane offer respective advantages and disadvantages and are widely used today. However, the lack of consistent clinical evidence and sparsity of RCTs limit the ability to conclusively regard propofol-based TIVA as a superior agent over inhalation anesthetics in reducing cancer recurrence and metastasis. More clinical research that covers a broader range of cancer subtypes is necessary to substantiate propofol’s superiority over sevoflurane regarding oncologic surgery. Additionally, POD and POCD remain significant challenges in postoperative care, especially in older adults undergoing major surgeries, and the role of anesthesia in promoting recovery must continue to be explored.

Given the millions of oncologic surgical cases expected in the coming decades within an increasingly younger population, it is crucial to work towards maximizing postoperative recovery and minimizing cancer recurrence and metastasis. Future large-scale, multicenter RCTs structured with long-term follow-up intervals and standardized cognitive assessments, and stratified by cancer subtype, are necessary to clarify the effects of anesthetics on oncologic surgery outcomes and recovery and provide more definitive guidance on anesthetic selection. Until more conclusive evidence emerges, the choice of anesthetic should be tailored to the individual patient, considering relevant comorbidities, patient-specific risk factors, tolerance of anesthesia, and pulmonary and cardiac stability.
